# Construction of a “Bacteria-Metabolites” Co-Expression Network to Clarify the Anti–Ulcerative Colitis Effect of Flavonoids of *Sophora flavescens* Aiton by Regulating the “Host–Microbe” Interaction

**DOI:** 10.3389/fphar.2021.710052

**Published:** 2021-10-14

**Authors:** Jing Shao, Zhaocheng Li, Yanping Gao, Kairui Zhao, Minling Lin, Yadi Li, Shumei Wang, Yi Liu, Lei Chen

**Affiliations:** ^1^ Key Laboratory of Digital Quality Evaluation of Chinese Materia Medical of State Administration of TCM, China, Engineering & Technology Research Center for Chinese Materia Medical Quality of Guangdong Province, School of Traditional Chinese Medicine, Guangdong Pharmaceutical University, Guangzhou, China; ^2^ The Fifth Affiliated Hospital of Southern Medical University, Guangzhou, China; ^3^ School of Chinese Medicine, Southern Medical University, Guangzhou, China

**Keywords:** ulcerative colitis, *16S rRNA* sequencing, gut bacteria, metabolomics, “host–microbe” interaction

## Abstract

Ulcerative colitis (UC) is considered an immune disease, which is related to the dysbiosis of intestinal microbiota and disorders of the host immune system and metabolism. *Sophora flavescens* Aiton has been used for the clinical treatment of UC in China and East Asia for thousands of years. It has many traditional prescriptions and modern preparations, and its curative effects are definite. We are the first to report that the flavonoids in *Sophora flavescens* (*S. flavescens*) Aiton EtOAc extract (SFE) could potentially attenuate the dextran sodium sulfate–induced UC in mice, which changed the current understanding of considering alkaloids as the only anti-UC pharmacological substances of *S. flavescens* Aiton. Based on the *16S rRNA* gene sequencing and metabolomic analysis, it was found that the anti-UC effects of SFE were due to the regulation of gut microbiota, reversing the abnormal metabolisms, and regulation of the short-chain fatty acids synthesis. Notably, according to the interaction networks of specific bacteria and “bacteria and metabolites” co-expression network, the SFE could enrich the abundance of the commensal bacterium *Lactobacillus*, *Roseburia*, *norank_f__Muribaculaceae*, *Anaerotruncus*, *Candidatus_Saccharimona*, and *Parasutterella*, which are proposed as potentially beneficial bacteria, thereby playing vital roles in the treatment of UC.

## Introduction

Ulcerative colitis (UC) is considered an immune disease, in which lesions mainly occur in the sigmoid colon and rectum, which are manifested by the inflammatory infiltration and injury of colonic mucosa. UC has a long course, a relapsing nature, and sometimes induces colorectal cancer. Epidemiological studies have shown that its global incidence has been increasing every year; it has been identified in the modern time as one of the hardest diseases to be cured and is known as “green cancer” (Ng et al., 2017; Ungaro et al., 2017; Danese et al., 2020). UC has a complex pathogenesis, which is attributed to the genetics, diet, immune abnormalities, and dysbiosis of intestinal microbiota (Liu and Stappenbeck, 2016; Ungaro et al., 2017; Khan et al., 2020). The changes in immunity, metabolism, and mucosal structure, caused by the “host–microbe” interaction, are the driving forces of UC. The gut microbiota has an intimate link with the host's health and disease and produces metabolites, which play an important role in this crosstalk. In UC, the diversity of intestinal microbiota and abundance of short-chain fatty acids (SCFAs)–producing and aromatic hydrocarbon receptors (AhRs)–producing bacteria decreased while that of the mucus-degrading and pathogenic bacteria increased, which caused changes in metabolites, such as butyric acid and 2-indolecarboxylic acid; the changes in these metabolites reduced their protective effect on the intestinal mucosa and affected the balance of Th and Treg cells, thereby accelerating the destruction of colonic mucosa and developing UC (Agus et al., 2018; Natividad et al., 2018; Nascimento et al., 2020). Hence, finding of drugs based on the regulation of “host–microbe” interaction might provide a novel therapeutic strategy for the treatment of UC.

“Kushen,” a well-known traditional Chinese medicinal herb stemmed from the dried roots of *Sophora flavescens* Aiton, belongs to the family *Leguminosae* and genus *Sophora* and has been used for the clinical treatment of UC alone or in combination with other drugs prescribed in ancient China (Ding et al., 2011; He et al., 2015; Cheng et al., 2016; Zhang et al., 2017). Our previous study found for the first time that the flavonoids in *Sophora flavescens* Aiton EtOAc extract (SFE) had a good therapeutic effect on UC rats (Chen et al., 2020a); these results also changed the current understanding of considering alkaloids as the only anti-UC pharmacological substances of *S. flavescens* Aiton (Cheng et al., 2006; Chen et al., 2017). On the other hand, the poor bioavailability of flavonoids (Yang et al., 2016) in SFE makes it difficult to support its significant pharmacodynamic activity; therefore, the mechanism of SFE in treating UC remains unclear. Our previous study showed that the flavonoids in SFE might become potential substrates for gut microbiota (Shao et al., 2020), suggesting that the SFE could improve UC by affecting the intestinal microbiota and adjusting the “host–microbe” interaction.

In this study, the pharmacodynamic effects of SFE on UC mice were demonstrated by investigating the structure and pathology of colonic tissue, expression of immune–inflammatory cytokines, and the levels of oxidative stress in colon tissue. The identification of microbiota was carried out using *16S rRNA* gene sequencing technology to understand the regulatory effects of SFE on intestinal microbiota of UC mice, to identify specific bacteria regulated by SFE, and to explore the relationships among the specific bacteria in intestinal microbiota. Metabolomics was also studied using ultra-high performance liquid chromatography–mass spectrometry (UHPLC-MS) technology to monitor the changes in the metabolites of UC and SFE-treated UC mice, and the differential metabolites were screened out. Based on the results of microbiome and metabolomics, the interaction network of specific bacteria and the co-expression network were combined to understand the relationship between metabolites and bacteria. The function-specific bacteria, which were regulated by the SFE during the treatment of UC, were screened out. These findings demonstrated the use of SFE against UC by regulating the “host–microbe” interaction.

## Materials and Methods

### Chemicals and Reagents

In October 2017, fresh *Sophora flavescens* (*S. flavescens*) Aiton root samples were harvested from the planting base of *S. flavescens* Aiton GAP in Chinese medicinal materials of Shanxi Zhendong Pharmaceutical Co., Ltd; Mesalazine was purchased from Heilongjiang Sunflower Pharmaceutical Group's Jiamusi Luling Pharmaceutical Co., Ltd; dextran sulfate sodium (DSS), benzidine, 3% H_2_O_2_, and chromatography grade ethyl acetate were purchased from Shanghai Macklin Biochemical Technology Co., Ltd; chromatography grade acetonitrile and formic acid were purchased from Thermo Scientific Co., Ltd; primary antibodies: IL-1β, IL-6, IL-10, TNF-α, COX-2, GADPH, and iNOS and secondary antibodies were purchased from Affinity Biosciences Co., Ltd; Kolliphor P 188 co-solvent was brought from BASF SE Co., Ltd; Distilled water was purchased from Watson Group Ltd. MOBIO PowerSoil® DNA Isolation Kit was purchased from MOBIO Co., Ltd; TransStart FastPfu DNA Polymerase was purchased from TransGen Biotech Co., Ltd; Quant-iT™ Broad-Range DNA Assay Kit and primer were purchased from Invitrogen Co., Ltd; agarose was purchased from Guangzhou Haoma Biological Technology Co., Ltd; superoxide dismutase (SOD), myeloperoxidase (MPO), and malondialdehyde (MDA) kits were purchased from Nanjing Jiancheng Bioengineering Institute; AxyPrep DNA Gel Extraction Kit was purchased from Axygen Co., Ltd; 2-methylvaleric acid was purchased from ChengduChroma-Biotech Co., Ltd; anhydrous sodium sulfate and sodium chloride powder were purchased from Beijing Yinuokai Technology Co., Ltd.

### Preparation and Determination of SFE

SFE was prepared following the procedures reported in our previous studies (Chen et al., 2020a). Briefly, the slices of *S. flavescens* Aiton were extracted three times for 2 h with 90% ethanol using reflux extraction method. The combined extracts were then evaporated, and the residues were reconstituted with warm water (1:8, v/v) and filtered. The aqueous solution was further extracted four times with ethyl acetate (1:1, v/v), which was then removed using a rotary evaporator, and the SFE obtained by lyophilization. The conditions for UHPLC-MS for the analysis of SFE are provided in the Supplementary Material 2.1.

### Animals Grouping and Drug Administration

A total of 45 male C57BL/6J mice, aged 6–8 weeks and weighing 18–22 g, were provided by the Experimental Animal Center of Southern Medical University [Guangzhou, China, Animal batch number: SYXK (yue) 2017-0125]. The mice were maintained in a room, having 22 ± 3°C temperature, 50 ± 10% humidity, and 12/12 h of light/dark cycle. The mice were divided into five groups (*n* = 9 per group), including the control, model, mesalazine, SFE-L, and SFE-H groups, after 1 week of adaptive feeding. The mice in the control group had access to water ad libitum for 10 days, and those in the other groups were given 2% DSS ad libitum for 10 days. During the experiment, the control and model groups were intragastrically administered with water; the mesalazine group was intragastrically administered with mesalazine (152 mg/kg, which was calculated based on the human dose used in clinics and then converted the human dose into mice dose according to the “human and laboratory animal dose conversion table”); and the SFE-L and SFE-H groups were intragastrically administered with 100 and 200 mg/kg of SFE, respectively [Kolliphor P 188 and deionized water (2:3) were used to dissolve SFE]. These group-specific diets were administered once daily for 10 consecutive days. The mice were deprived of food the night before the end of the experiment. Their colons were removed and the intestinal contents and colon tissues were collected before anesthetized, which were then cleaned using the precooled normal saline. The tissues and intestinal contents were stored at −80°C until further use.

### Determination of Pharmacodynamic Effects of SFE

During the experimental process, the mental state and weight of the mice were observed and measured daily at the same time, the mental state of the mice was evaluated by observing the claw force and the number of times the mice climbed over the cage within an hour when the mice were active in morning, as well as food intake, and the colon lengths were measured after removal. For the evaluation of colon permeability, the detailed standard protocols were obtained from previous studies (Li et al., 1994; Kitajima et al., 1999; Liu, 2009) and the JoVE experiment video (https://www.jove.com/); the detection of cyclooxygenase-2 (COX-2) and inducible nitric oxide synthase (iNOS) in the oxidative stress evaluation was carried out using Western blot analysis; their detailed standard protocols are provided in the Supplementary Material. In the oxidative stress evaluation, the activity of MPO, SOD, and MDA was detected using MPO colorimetric activity assay kit, SOD assay kit (WST-1 method), and MDA assay kit (TBA method), respectively. The tissue samples for the measurement of MPO, SOD, and MDA activities were pretreated as follows; an appropriate amount of the colon tissues were weighed, to which normal saline was added (9:1) for homogenization. The tissue samples for the measurement of COX-2 and iNOS activities were pretreated as follows: an appropriate amount of the colon tissue was added into 500 µl of lysate (protease inhibitor: RIPA lysate mixed at ratio of 1:500) to fully homogenize, kept for 30 min on ice, and then centrifuged at 12,000 rpm/min for 15 min to obtain the supernatant.

For the histopathology, immunofluorescence (IF), and immunohistochemistry (IHC) analyses, the cleaned colonic tissue samples were immersed in 4% paraformaldehyde for 24 h. The detailed procedure has been provided in the Supplementary Material. The histopathological damage of the colon was evaluated according to the Sykes score (Takagi et al., 2011) and the number of goblet cells were counted (six fields of view were selected for each slice to calculate the number of goblet cells) as described previously (Shi et al., 2015; Ke, 2020) using Image J (National Institutes of Health Co., Ltd) after hematoxylin and eosin staining. The cleaned colon tissue samples were then embedded in paraffin and sliced. The CD4^+^ T cells were detected using the IF assay. The levels of IL-1β, IL-6, IL-10, and TNF-α were detected and quantified using the IHC assay and IPP software (Media Cybernetics Co., Ltd).

### *16S rRNA* Gene Sequencing and Interaction Network Analysis of Specific Bacteria

The metagenomic DNA from the intestinal contents was extracted using the DNA kit and detected using agarose gel electrophoresis. The *16S rRNA* genes were amplified with a specific primer pair using PCR instrument. The PCR products were mixed in equal ratios. Then, the mixture of the PCR products was purified using the Gel Extraction Kit. The sequencing libraries were generated using the TruSeqTM DNA Sample Prep Kit following the manufacturer's instructions, and the index codes were added. DNA libraries were loaded and sequenced on an Illumina Hiseq 2500 platform.

The raw mate-paired FASTQ files were quality filtered and demultiplexed, followed by the sequences analysis. The sequences with more than 97% similarities were clustered into the same operational taxonomic units (OTUs), and the OTU classification at each level was carried out according to the database. The OTUs were sorted out by the number of sequences that each OTU contained in each sample. In the rank-abundance curve, the ranking levels acted as abscissa and the number of sequences of each OTU acted as the ordinate. The differences among the groups were analyzed using Alpha diversity index. A rarefaction curve was constructed based on the number of sequences and their corresponding OTU numbers or diversity index. The composition of intestinal microbiota in each sample was analyzed at the level of phylum, class, order, family, and genus. The bacteria with greater abundance were selected to draw the community bar and HeatMap. The hierarchical cluster analysis of each sample and the principal coordinate analyses (PCoA) of the beta diversity analysis were performed. The type of bacteria was analyzed based on the Bray–Curtis clustering method. The specific bacteria were screened out using LEfSe analysis. In order to analyze the relationship among the different bacteria, Spearman's correlation analysis was performed on the top 50 bacteria at the genus level. The bacteria that showed a strong correlation with other specific bacteria were screened out and imported into the Cytoscape 3.7.2 software (https://cytoscape.org/) to construct a bacterial interaction network. The metabolic functional annotation of each OTU was obtained using PICRUSt analysis (phylogenetic investigation of communities by reconstruction of unobserved state); the pathways information was obtained according to the KEGG database, and the abundance of each functional category was calculated according to the OTU abundance. Finally, the data were analyzed using the STAMP software (http://kiwi.cs.dal.ca/Software/STAMP) for the analyses of differences between the groups and data visualization.

### Metabolomics Analysis

The intestinal contents were freeze-dried overnight, and 10 mg of the freeze-dried powder of each sample was accurately weighed, to which 135 μl of acetonitrile and 45 μl of distilled water were added. The intestinal contents were homogenized using sonication at 60 Hz for 10 min. After centrifugation at 12,000 rpm and 4°C temperature for 15 min, the supernatant was transferred to a sample vial for UHPLC-MS analysis. The metabolomics analysis was performed using an Ultimate 3000 LC system coupled with an Exactive Quadrupole-Orbitrap–Mass Spectrometry and an electronspray ionization source (Thermo Fisher Scientific, Germany). The liquid chromatographic separation for the processed intestinal contents was achieved using an ACQUITY BEH C18 column (1.7 μm, 2.1 mm × 100 mm, Waters, United States). The detailed conditions for the chromatography and mass spectrometer are provided in the Supplementary Material, which was similar to our previous study (Shao et al., 2020).

The Compound Discoverer 3.0 software (Thermo Fisher Scientific) was used to identify, extract, and correct the raw metabolomics data to obtain the retention time, precise molecular weight, and peak area of each sample. SIMCA-P 14.1 software was used to establish the principal component analysis (PCA) and orthogonal partial least squares discrimination analysis (OPLS-DA) models for the control and model groups and to filter out the variables with importance projection value (VIP) > 1, fold change (FC) > 2 or < 0.5, and *p*-value < 0.05. In the model and SFE-H groups, the variables with *p*-value < 0.05 and FC > 2 or < 0.5 among the selected variables were filtered out, which were the differential variables of the untargeted intestinal content metabolomics analysis. The FC > 2 and < 0.05 indicated that the metabolite was upregulated and downregulated, respectively.

### Determination of SCFAs

The SCFAs quantification method has been described in our previous study (Wang et al., 2020). Briefly, 10 mg of the powdered intestinal content was accurately weighed and 400 μl of the precooled saturated sodium chloride solution and appropriate internal standard were added to make the concentration of internal standard as 10 μg/ml. 5 μl of 10% H_2_SO_4_ was added to acidify the system. The sample was homogenized for 10 min and 400 μl of the precooled ethyl acetate was added to it, which was then again homogenized for 10 min and centrifuged at 12,000 rpm and 4°C temperature for 10 min. The supernatant was obtained, and 0.25 g of the anhydrous sodium sulfate was added to the supernatant and centrifuged at 12,000 rpm and 4°C for 10 min. The supernatant was obtained for gas chromatography–mass spectrometry (GC-MS) detection. The determination of SCFAs was performed on 7890B gas chromatography coupled with a 7000D mass spectrometric detector (Agilent Technologies, United States). The SCFAs were separated using a DB-FFAP column (30 m × 0.25 mm, 0.25 μm). The detailed chromatography and mass spectrum conditions are provided in the Supplementary Material. The Agilent MassHunter quantitative analysis software was used to determine the contents of SCFAs in each sample by comparing the peak area of SCFAs and internal standards in each sample.

### Establishment of Co-Expression Network and the Evaluation of Functional Bacteria

The effects of SFE regulation on the specific intestinal microbiota in UC mice were obtained using the *16S rRNA* gene sequencing analysis. The differential metabolites were obtained according to the metabolomics analysis, including the metabolic pathways. Using the PICRUSt analysis, the overlapped metabolic pathways between the control–model and model–SFE-H were obtained, these metabolic pathways overlapped with the metabolomics pathways. Finally, the metabolites involved in the overlapped metabolic pathways were selected. Spearman's correlation analysis was performed for the peak areas of the selected metabolites, SCFAs, and relative sequence numbers of the specific bacteria (wkomics.omicsolution.com). A co-expression network was constructed using Cytoscape 3.7.2 software (interactions in the network had *p*-values < 0.05 and coefficients > 0.5). The functionally specific bacteria related to the development of UC and SFE treatment were screened using the co-expression and interaction networks of the specific bacteria. Finally, the ROC curves were established to evaluate the functional specific bacteria.

### Statistical Analyses

The experimental data were expressed as mean ± standard deviation (x ± SD) and analyzed with GraphPad prism (https://www.graphpad.com/scientific-software/prism/) using one-way analysis of variance (ANOVA). In LEfSe analysis, the nonparametric factorial Kruskal–Wallis (K-W) rank-sum test was used to analyze the bacteria with significant differences in abundance among the different groups. The Wilcoxon rank-sum test was used to analyze the differences between the groups. Finally, the LDA effect size analysis was performed. In PICRUSt analysis, Welch's analysis was used to analyze the significant differences in the metabolic function of bacteria among the different groups. The differences in the abundances of metabolites between the two groups were analyzed using the Mann–Whitney test.

## Results

### Result of SFE Determination

**FIGURE 1:**
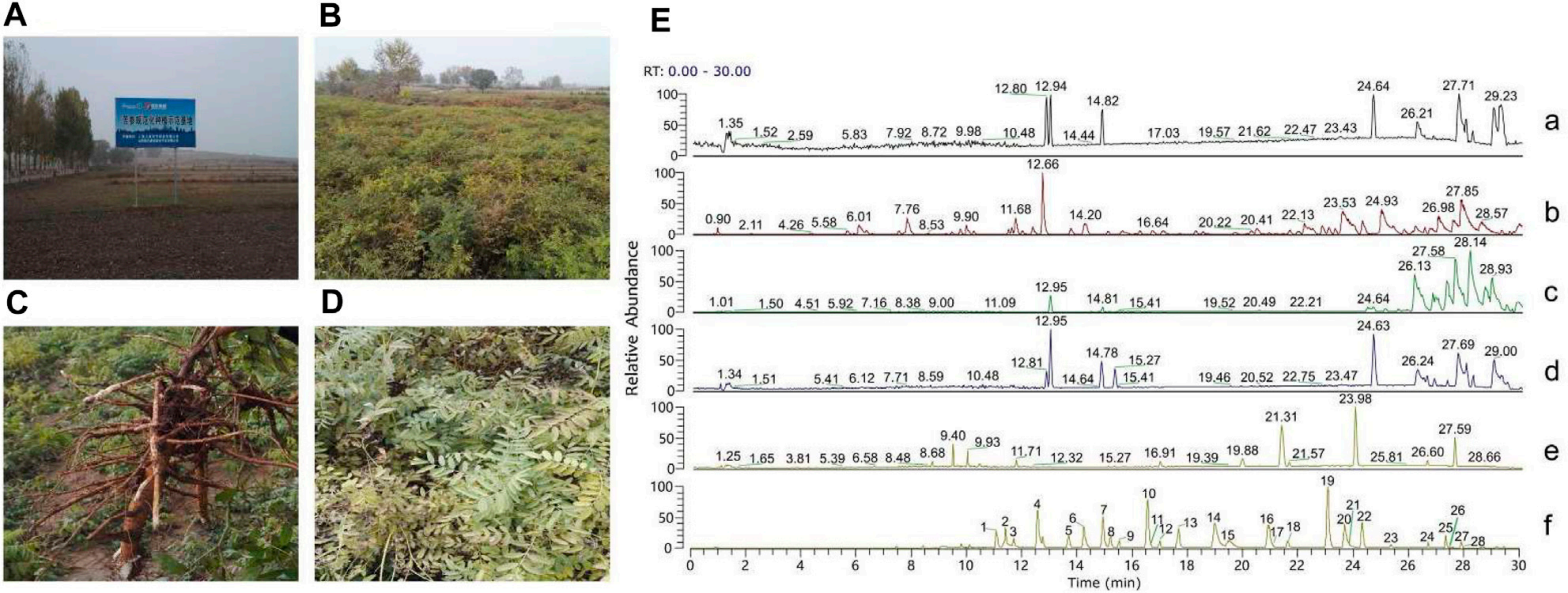
**(A)** The GAP planting base (N 36°33′20″, E 113°03′55″) of *Sophora flavescens* Aiton. **(B)** Fields for planting *S. flavescens* Aiton. **(C)** The root morphology of *S. flavescens* Aiton. **(D)** The leaf morphology of *S. flavescens* Aiton. **(E)** Total ion chromatograms of **(a)** blank plasma, **(b)** plasma sample after 45 min SFE administration, **(c)** blank urine, **(d)** urine sample for 0–24 h after SFE administration, **(e)** SFE, and **(f)** standard references.

The *S. flavescens* Aiton samples were identified by Dr. Lei Chen (College of Traditional Chinese Medicine, Guangdong Pharmaceutical University). The collection locations and characteristics of *S. flavescens* Aiton samples are shown in Figures 1A–D. A voucher (20171025) was deposited at the College of Traditional Chinese Materia Medica, Guangdong Pharmaceutical University. The identification of SFE and their prototypes in rat urine and plasma were demonstrated in our previous study (Chen et al., 2020a), which is shown in Figure 1E, and the detailed information of the flavonoids is provided in Supplementary Table S1. Furthermore, the contents of the four representative compounds, including kurarinone, norkurarinone, kushenol N, and kushenol L, in the SFE were 63.2, 35.3, 23.7, and 1.9 mg/g, respectively (Chen et al., 2019), which were determined using UHPLC-MS and are provided in Supplementary Table S2.

### SFE Effect on the Structure of Colon, Oxidative Stress Level, the Expression of Immune Inflammatory Cytokines, and the Number of Immune Cells

**FIGURE 2:**
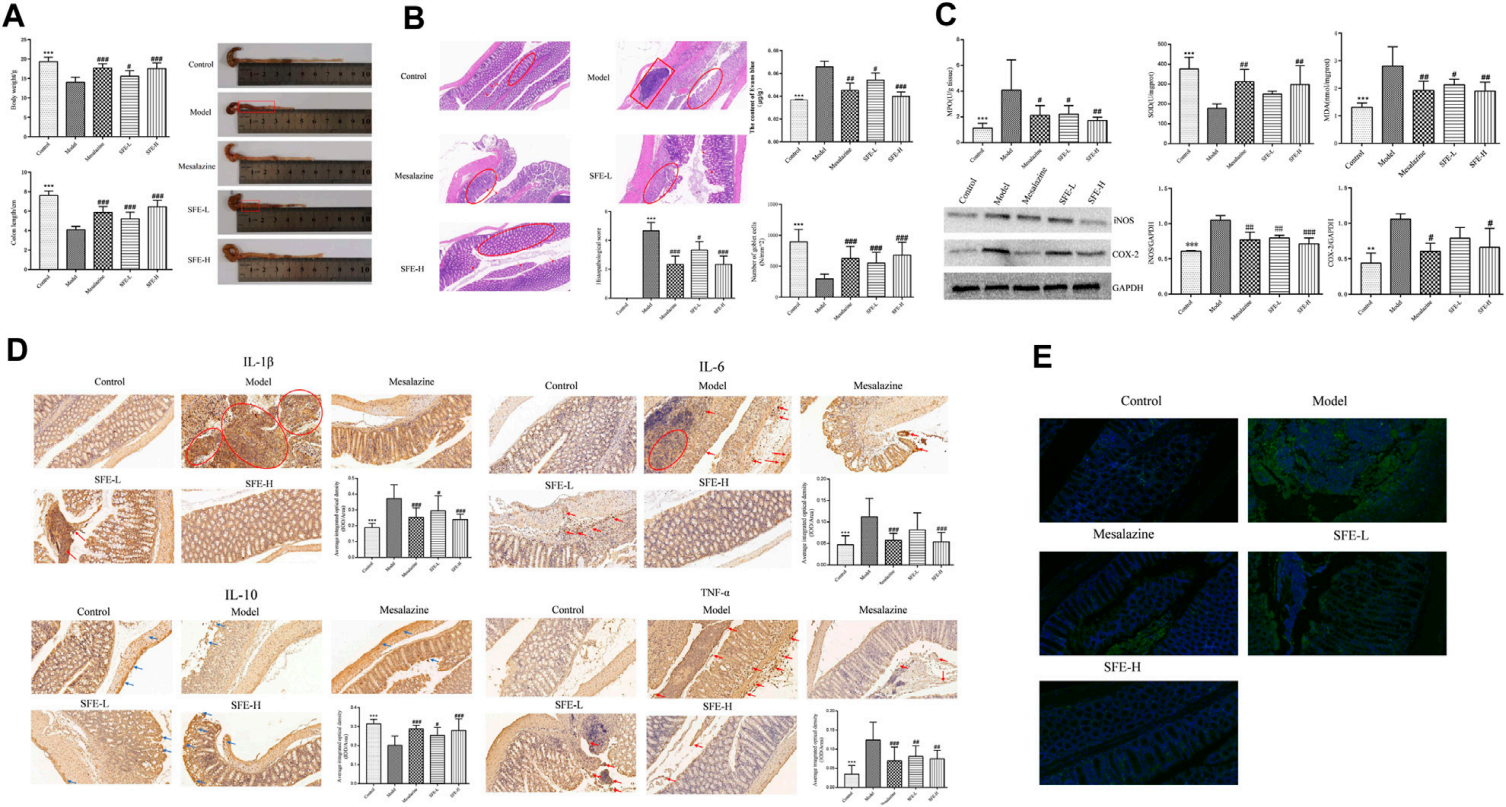
SFE treatment alleviated DSS-induced UC. **(A)** The body weight and colon length of mice. **(B)** The pathological slice of mice (×400 magnification), pathological score, the number of goblet cells, and the content of Evans blue of mice. **(C)** The activity of MPO and SOD, the content of MDA, and the expression of iNOS and COX-2 in the colon tissue of the mice. **(D)** The immunohistochemical slice of the mice that express the secretion of IL-1β, IL-6, IL-10, and TNF-α, and their average integrated optical density of the cells. **(E)** The investigation of CD4^+^ T cells in the colon tissue of mice by immunofluorescence. * means that the control group compared with the model group is significant; # means that the model group compared with the administrated group is significant; *, # means *p* < 0.05; **, ## means *p* < 0.01; ***, ### means *p* < 0.001.

The investigation of the efficacy of SFE showed that it had notable therapeutic effects in treating UC, which were dose-dependent. As compared to the control group, the body weight and colon length of the mice in the model group were significantly reduced, and their colons also showed significant swelling (Figure 2A, represented by the red squares). The SFE effectively relieved the weight loss and colon atrophy in UC mice. The therapeutic effect of SFE-H was equivalent to or better than that of the mesalazine (Figure 2A). The UC mice showed deformed, necrotic, and shedding epithelium, while the crypt and goblet cells disappeared and the lymphocytes were diffusely aggregated in the basal (rectangular) area. In contrast, the SFE-H group had significantly repaired mucosa, no tissue necrosis and shedding, a small number of aggregated lymphocytes, tight arrangement of the crypt (circled area), and increased number of goblet cells. The content of Evans blue in the colonic tissue of the SFE-L, SFE-H, and UC mice groups were 0.054 ± 0.0062, 0.039 ± 0.0039, and 0.067 ± 0.0049 μg/g, respectively, indicating the repairing effects of SFE on mucosal permeability. The histopathologic analysis and evaluation of colon permeability can be seen in Figure 2B.

According to the oxidative stress evaluation of the mice (Figure 2C), the MPO activity of the UC mice increased, while that in the SFE-H group showed reduced activity of MPO in the model group and its effects were better than mesalazine. The SFE-H could reverse the decrease in SOD and increase in the MDA activities significantly, which is comparable to the mesalazine. The expression levels of COX-2 and iNOS increased significantly in the UC mice, which decreased after the administration of SFE.

In the IHC analysis (Figure 2D), the deep brown color, indicated by the red arrows and circled, is the positive expression of IL-1β, IL-6, IL-10, and TNF-α in the colon tissue. The positive expression of IL-1β, IL-6, and TNF-α showed aggregated clumps in the mucosal layer of the colon and diffused spots in the muscle layer. The SFE reduced the expression of these cytokines in mucosal and muscular layers to varying degrees. The secretion of IL-10 in the colon tissue of the model group was insufficient (indicated by the blue arrows) with light brown color, which changed to dark brown after SFE administration, suggesting an increase in its secretion; as shown by IF (Figure 2E), light green was observed in CD4^+^ cells in the colon tissues. The number of CD4^+^ T cells in the colon tissue of the UC mice increased sharply, which was reduced greatly after the administration of SFE.

In conclusion, the SFE was found to effectively alleviate the weight loss of UC mice, repair the structure of colon tissue, and reduce oxidative stress level and immune inflammation in UC mice, which could be observed from the length of the colon, pathology of the tissue, colon permeability, the number of goblet cells, the level of oxidative stress, the expression of immune–inflammatory factors, and the number of immune cells.

### SFE Reduced Pathogenic Bacteria, Increased Beneficial Bacteria, and Restored the Normal Structure of Intestinal Microbiota in UC Mice

**FIGURE 3:**
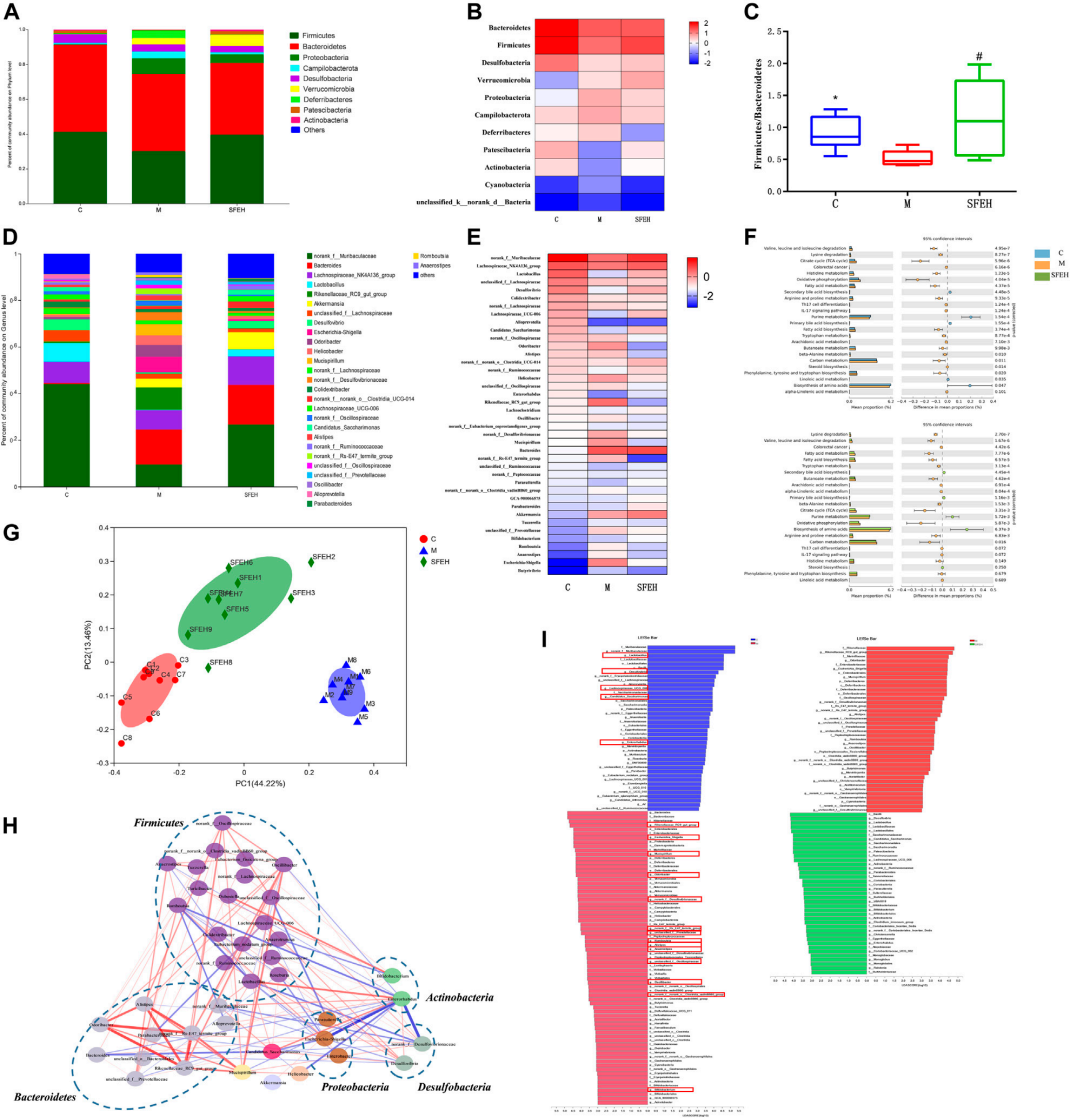
SFE alleviated DSS-induced gut dysbiosis. **(A,D)** The community bar of the control, model, and SFE-H groups at the phylum and genus levels. **(B,E)** The community HeatMap of the control, model and SFE-H group at the phylum and genus levels; **(C)** *Firmicutes*/*Bacteroidetes* value of each group; **(F)** PICRUSt analysis between the control and model groups, and between the model and SFE-H groups. **(G)** The PCoA model of the control, model, and SFE-H groups based on the Bray–Curtis algorithm. **(H)** The interaction network of the specific bacteria (the strength of the interaction is represented by the thickness and depth of the lines. The stronger the interaction, the thicker and darker a line is. The red line represents the positive relationship, and the blue line represents the negative relationship). **(I)** The LDA scores of discriminative taxa in the control and model groups, and in the SFE-H and model groups. * means that the control group compared with the model group is significant; # means that the model group compared with the administrated group is significant; *, # means *p* < 0.05; C, M, and SFE-H represent the control, model, and SFE-H groups, respectively.

The intestinal microbiota in all the samples exhibited 1160 OTUs. The rank abundance curve proved that the abundance of species in the samples was sufficient and showed the homogenous distribution of species (Supplementary Figure S1A). The Alpha diversity analysis showed that there were no significant differences in species diversity among the control, model, and SFE-H groups (Supplementary Figure S1B). The rarefaction curve of each sample tended to be flat with the increase in the number of sequences, which indicated the sequencing depth was sufficient for each sample (Supplementary Figure S1C). The hierarchical cluster analysis of beta-diversity showed that the SFE-H and control groups were in the same cluster, suggesting that the bacteria structure of the SFE-treated mice was similar to that of the control mice (Supplementary Figure S1D). When beta-diversity was analyzed using the Bray–Curtis dissimilarity metric in PCoA and bacteria typing analysis, clear segregation of the gut microbial signatures between the control and model groups was observed. The distance between the SFE-H and control groups was shorter than that of the control and model groups (Figure 3G). In the bacteria typing analysis, most of the samples in the SFE-H group and all the samples in the control group were clustered into a similar category, while those of the model group were clustered into another category (Supplementary Figure S1E).

As compared to the control mice, the abundance of the dominant phylum, which included *Proteobacteria*, *Campilobacterota*, *Deferribacteres*, and *Verrucomicrobia*, were upregulated in the UC mice, while the phylum *Desulfobacteria*, *Patescibacteria*, and *Actinobacteria* were downregulated. It was worth noting that the abundances of bacteria in the SFE-H group at the phylum level were similar to that of the control mice except for the phylum *Verrucomicrobia* (Figures 3A,B). The ratio of *Firmicutes* to *Bacteroidetes* (F/B) was reversed by SFE, which had declined in the UC mice because of the significant decrease in the abundance of *Firmicutes*, while *Bacteroidetes* did not change sharply (Figure 3C). Consistent with the results at the phylum level, the SFE reversed the decreasing trend of the genus *norank_f__Muribaculaceae* (*norank_f__Bacteroidales_ S24_7_group*), *Lachnospiraceae_ NK4A136_group*, *Lactobacillus*, *unclassified_f__ Lachnospiraceae*, *Desulfovibrio*, *norank_f__Lachnospiraceae*, *Colidextribacter*, and *Lachnospiraceae_UCG*-006 in UC mice, which were the most abundant bacterial genera in the control mice, accounting for about 80% of the intestinal microbiota. On the other hand, the SFE eliminated the pathogenic bacteria, including *Rikenellaceae_RC9_gut_group*, *Escherichia–Shigella*, *Odoribacter*, *Helicobacter*, *Mucispirillum*, *norank_f__Desulfovibrionaceae*, *Alistipes*, and *norank_f__Rs-E47_termite_group* in UC mice and promoted the abundance of bacteria that were dominant in the control mice (Figures 3D,E).

LEfSe analysis of the taxonomic alternations revealed that UC and SFE elicited unique sets of bacterial enrichment or deficiency. In the LEfSe analysis, the bacteria that had an LDA score of >3 between groups were selected for further analysis. In the control and model groups, there were 111 bacteria with significant differences; in the SFE-H and model groups, 79 bacteria had significant differences. The results of the model–control groups and the SFE-H–model groups were overlapped, and it was found that SFE-H could reverse 55 bacteria that had significant changes in the UC mice; these bacteria were focused to the genus level; 19 specific bacteria that were closely related to the development of UC and the therapeutic effect of the SFE were obtained, and they were framed in red squares in Figure 3I.

### Interaction Network of Specific Bacteria in the Onset of UC and SFE Treatment and their Metabolic Functions

Spearman analysis of the genus that was shown on HeatMap was performed. The bacteria that had a strong relationship with the 19 selected bacteria in the LEfSe analysis were screened out (the absolute value of the correlation coefficient was greater than 0.6, and the *p*-value was less than 0.05). Finally, 21 bacteria were screened out and an interaction network was constructed for these 40 differentiated bacteria, which is shown in Figure 3H.

The genera *Lactobacillus*, *Roseburia*, *Parasutterella*, *norank_f__Muribaculaceae*, and *Candidatus_Saccharimonas* decreased in UC mice and increased after the SFE-H treatment. These bacteria had the following strong relationships: among them, the genus *Lactobacillus* had a strong negative correlation with *Romboutsia*, *norank_f__Desulfovibrionaceae*, and *Escherichia–Shigella* and had a strong positive interaction with *Enterorhabdus*, *norank_f__Muribaculaceae*, *Candidatus_Saccharimonas*, and *Alloprevotella*. The genus *Roseburia* had a strong negative interaction with *Escherichia–Shigella* and *Romboutsia* and had a strong positive interaction with *Candidatus_Saccharimonas*. The genus *Parasutterella* had a strong negative correlation with *unclassified_f__Oscillospiraceae*, *Alistipes*, and *Rikenellaceae_RC9_gut_group*. The genus *norank_f__Muribaculaceae* had a strong negative correlation with *norank_f__Desulfovibrionaceae*, *Romboutsia*, and *Escherichia–Shigella* and had a strong positive correlation with *Enterorhabdus*, *Candidatus*, and *Saccharimonas*. The genus *Candidatus_Saccharimonas* had a strong negative correlation with *Bacteroides*, *Escherichia-Shigella*, *norank_f__Desulfovibrionaceae*, and *Helicobacter* and had a strong positive correlation with *Desulfovibrio.* Among the bacteria with increased abundance in the UC mice and which decreased after SFE-H treatment, the genera *Alipites*, *norank_f__Rs-E47_termite_group*, *Rikenellaceae_RC9_gut_group*, *Odoribacter*, *Mucispirillum*, and *norank_f__Oscillospiraceae* had a strong positive interaction with each other. The genus *Romboutsia* had a strong positive interaction with *Escherichia–Shigella* and *unclassified_f__Prevotellaceae.*

In this study, the PICRUSt analysis was used to predict the metabolic function of intestinal microbiota. In UC, the structure and abundance of intestinal microbiota were altered, which led to the increased ability of the bacteria involved in most of the amino acid metabolism, immune–inflammatory response, lipid metabolism, and energy metabolism, while significantly decreased the ability of bacteria involved in the bile acid biosynthesis and purine metabolisms, such as arginine and proline metabolism, beta-alanine metabolism, valine, leucine, isoleucine degradation, arachidonic acid metabolism, fatty acid metabolism and synthesis, tricarboxylic acid cycle, and oxidative phosphorylation. After the SFE treatment, the metabolic function of dominant bacteria in the UC mice tended to reduce the amino acid and energy metabolism and increased the bile acid and purine metabolism (Figure 3F).

### SFE Reverse DSS-Induced Abnormal Energy, Amino Acid, Bile Acid, and Fatty Acid Metabolism

The UHPLC-MS was successfully used for metabolomics. The total ion chromatogram of QC is provided in Supplementary Figure S2A. The retention time and peak intensity of the ions in the positive and negative modes showed acceptable relative standard deviation (Supplementary Table S5). The above results confirmed the stability and repeatability of the LC-MS system, which provides reliable results for the metabolomics analysis.

**FIGURE 4:**
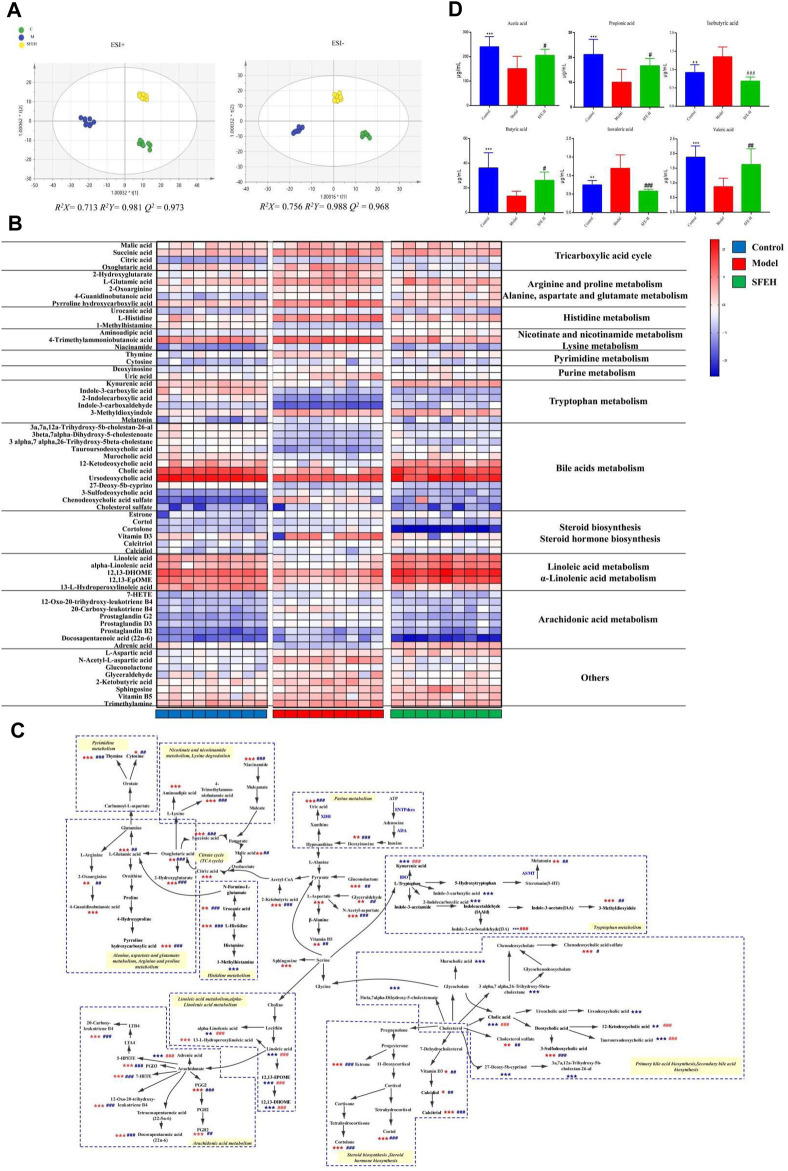
SFE reversed DSS-induced abnormal metabolism. **(A)** OPLS-DA score of the control, model, and SFE-H groups and QC in positive and negative modes, respectively. **(B)** HeatMaps of the differential metabolites that were altered by DSS feeding and SFE treatment. **(C)** Metabolic pathway network, the significant labels with blue color represent that the metabolite is downregulated, and those with red color represent that the metabolite is upregulated. **(D)** The concentration of SCFAs in the intestinal contents of the control, model, and SFE-H groups. * means that the control group compared with the model group is significant; # means that the model group compared with the administrated group is significant; *, # means *p* < 0.05; **, ## means *p* < 0.01; ***, ### means *p* < 0.001. C, M, SFE-H, and QC represent the control, model, SFE-H, and quality control groups, respectively.

The distinct clustering of metabolites was apparent among the control, model, and SFE-H groups as seen in the PCA in the positive and negative modes (Supplementary Figure S2B). The OPLS-DA also showed distinction among these groups (Figure 4A). The indicators *R* ^*2*^ *X*, *R* ^*2*^ *Y*, and *Q* (Danese et al., 2020) and the response permutation testing (Supplementary Figure S2C) indicated that the stability and predictability of OPLS-DA were reliable. The PCA and OPLS-DA models indicated that there were significant differences between the metabolic profiling of the control and UC mice, whereas those of the UC group were restored to normalcy gradually after the SFE treatment. The identification of differential variables was carried out as described in our previous study (Shao et al., 2020), and differential metabolites were analyzed for their role in metabolic pathways using the KEGG and MetaboAnalyst databases, SMPDB, and literature studies. The DSS triggered widespread change in 64 metabolites in the administered groups as compared to the control mice, which were partially reversed by SFE treatment (Supplementary Table S6 and Figure 4B). These metabolites can be involved in the metabolic pathways shown by the blue dotted box in Figure 4C, such as the tricarboxylic acid cycle (TCA) cycle, tryptophan metabolism, and arachidonic acid metabolism.

The levels of SCFAs in the intestinal content of each group were detected using GC-MS, which showed an increase in the levels of isobutyric acid and isovaleric acid, while that of acetic acid, propionic acid, butyric acid, and valeric acid decreased in the model group. However, their levels were reversed after the SFE treatment (Supplementary Figure S2D, Figure 4D).

### “Bacteria-Metabolites” Co-Expression Metabolic Network Regulated by SFE and the Functional Specific Bacteria

**FIGURE 5:**
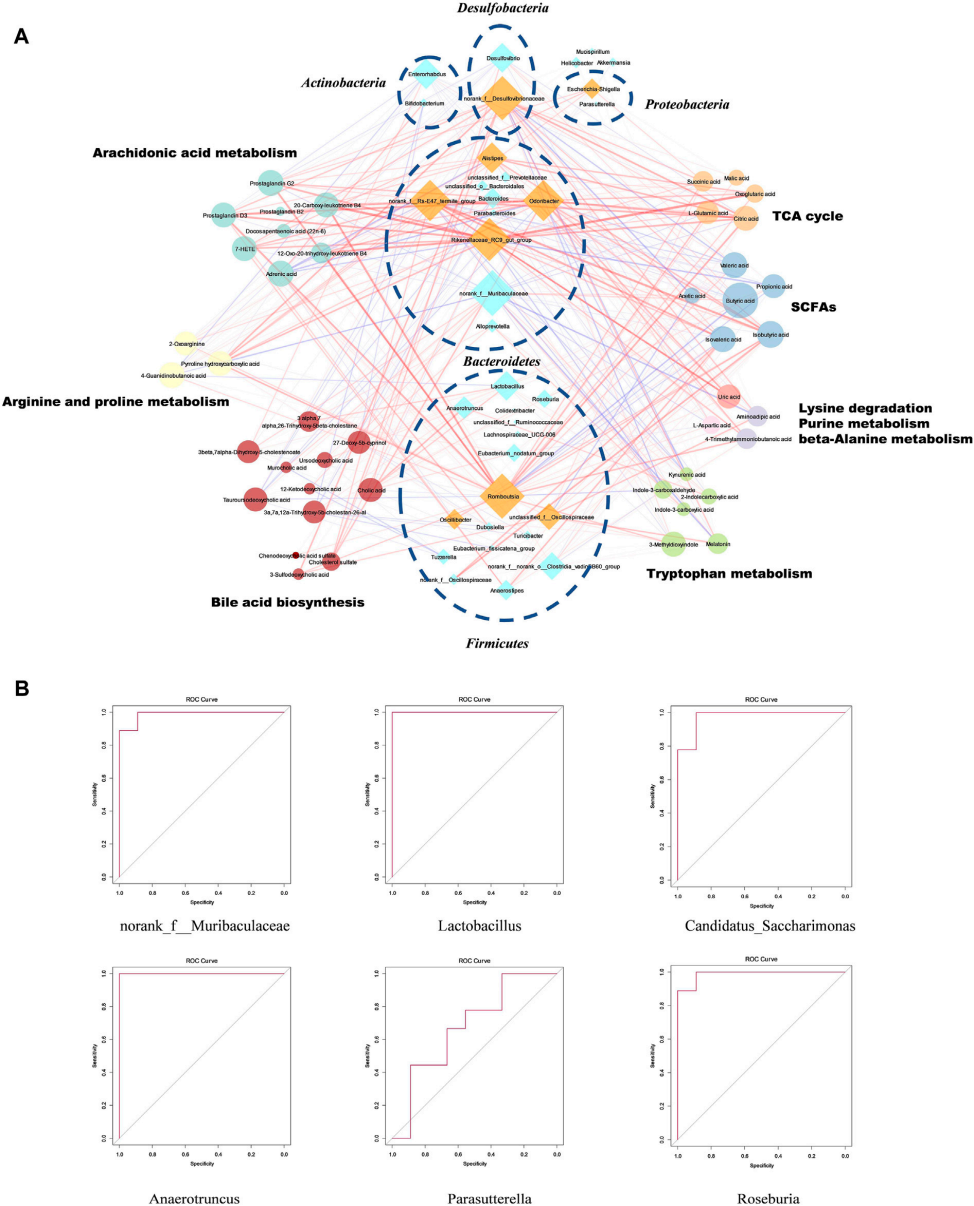
The relationship between bacteria and metabolic pathways, and functional specific bacteria in SFE treatment. **(A)** Co-expression network of specific bacteria and differential metabolites (the strength of the interaction is represented by the thickness and depth of the lines. The stronger the interaction, the thicker and darker a line is. The red line represents the positive relationship, and the blue line represents the negative relationship, the main pathogenic bacteria of UC are shown as orange). **(B)** The ROC curve constructed based on six functional specific bacteria.

The overlapped metabolic pathways of the host and intestinal microbiota included TCA cycle, lysine degradation, bile acid synthesis (primary and secondary bile acids), arginine and proline metabolism, beta-alanine metabolism, tryptophan metabolism, purine metabolism, and arachidonic acid metabolism. A total of 38 metabolites were involved in these pathways (Figure 5A).

The pathways of lipid metabolism in the co-expression network included arachidonic acid and bile acid metabolisms. The co-expression network showed that the arachidonic acid metabolism had strong positive correlations with the genera *Odoribacter*, *norank_f__Rs-E47_termite_group*, *Rikenellaceae_RC9_gut_group*, and *Romboutsia*, indicating that the abovementioned bacteria could enrich the metabolites involved in the arachidonic acid pathway. Free bile acids had strong positive correlations with *Lactobacillus*, *norank_f__Muribaculaceae*, and *Anaerotruncus*, suggesting that these bacteria were conducive to the decomposition of bile acids or increased the expression of the farnesoid X receptor (FXR), while the sulfurized bile acids had strong positive correlations with *Romboutsia* and *norank_f__Desulfovibrionaceae*, suggesting that they might promote the sulfidation of free bile acids. The co-expression network also showed the correlations among amino acid metabolism, SCFAs metabolism, and bacteria. The indole derivatives (AhR ligands) in tryptophan metabolism had a significant positive correlation with *Lactobacillus*, *norank_f__Muribaculaceae*, and *Alloprevotella*, suggesting that they might be AhR agonists or could promote the synthesis of AhR ligands. In the co-expression network of SCFAs and other metabolites involved in the TCA cycle, the SCFAs were positively correlated with *norank_f_Muribaculaceae*, *Roseburia*, and *Lactobacillus*, indicating that they could synthesize SCFAs. The metabolites involved in the TCA cycle were positively correlated with *Odoribacter*, *Rikenellaceae_RC9_gut_group*, *norank_f_Desulfovibrionaceae*, *norank_f_Rs-E47_termite_group*, and *Romboutsia*, indicating that they could consume their energy by destroying the intestinal mucosa and by consuming SCFAs. The metabolism of arginine, proline, and glutamate is related to oxidative stress in the intestine and had strong positive correlations with *norank_f__Desulfovibrionaceae*, *Romboutsia*, and *Rikenellaceae_RC9_gut_group*, while having strong negative correlations with *norank_f__Muribaculaceae*, indicating that the *norank_f__Muribaculaceae* might inhibit oxidative stress.

According to the co-expression and interaction networks of specific bacteria, the genera *Anaerotruncus*, *Lactobacillus*, *norank_f__Muribaculaceae*, *Roseburia, Candidatus_Saccharimonas*, and *Parasutterella* were speculated to be functionally specific bacteria, which might play therapeutic roles in the development of SFE as a treatment strategy for UC. As shown in Figure 5B, the ROC curves were constructed to evaluate and diagnose the functionally specific bacteria. The areas under the ROC curves of *Anaerotruncus*, *Lactobacillus*, *norank_f__Muribaculaceae*, *Roseburia*, *Candidatus_Saccharimonas*, and *Parasutterella* were 1, 1, 0.9877, 0.9877, 0.9753, and 0.679, respectively. The above results indicated that in the limited samples of our study, these six bacteria can be used as specific bacteria to distinguish between the healthy and UC mice. SFE might play an important role in the treatment of UC by increasing the abundance of these six bacterial genera, but this conclusion needs to be further verified in a larger sample size.

## Discussion

When UC happened, the physiological and pathological status of the mice were changed. The summary of the known physiology and pathology of UC and the interactions between the pathological responses are shown in Supplementary Table S7, and SFE can alleviate the pathology process and regulate these interactions to play a therapeutic role in UC. The number of goblet and epithelial cells is reduced, destroying their functions during UC. Therefore, the mucosal barrier gets broken. Then, the immune inflammation in lamina propria is induced by the invasion and destruction of harmful dietary antigens and pathogens, triggering oxidative stress (Sands, 2015; Huang et al., 2020; Ke, 2020; Zhao et al., 2020). Oxidative stress and immune inflammation affect each other and aggravate the pathological process of UC together. In the UC mice, the level of oxidative stress increased, while the SOD activity decreased, which plays the role of scavenging free radicals, thereby triggering the lipid peroxidation to form lipid peroxides (MDA activities). The neutrophils inflammatory infiltration, indicated by MPO activity (Li, 2019; Shen, 2019; Vaccaro et al., 2020), COX-2, and iNOS, has also been reported to promote oxidative damage and mediate the cellular anti-inflammatory and antioxidant signaling pathway in the host (Liu et al., 2011; Zhang, 2012; Li et al., 2014a). On the other hand, under oxidative stress, glutathione is increased as an antioxidant, and its precursor glutamate reacts with reactive oxygen species (ROS) until glutamate is depleted (Bjerrum et al., 2010; Morgan et al., 2012). The bile acids accumulate in the liver during the progression of UC, combine with the cell membranes to release arachidonic acid, and promote the generation of ROS, thereby aggravating oxidative stress and inducing DNA damage and liver cancer (Sánchez, 2018). In this study, the levels of glutamate, 20-carboxy-leukotriene B4, 12-oxo-20-trihydroxy-leukotriene B4, and prostaglandins PGG2, PGB2, and PGD3 increased significantly in the UC mice. Among these, the 20-carboxy-leukotriene B4 and 12-oxo-20-trihydroxy-leukotriene B4 promoted the production of IL-1, IL-6, IL-4, and prostaglandin under the action of COX-2 (Zhang and Wan, 2008; Zheng et al., 2011). On the other hand, the α-linolenic acid decreased in the UC mice. It is an omega-3 unsaturated fatty acid with anti-inflammatory and antioxidant effects (Kim et al., 2014), and is reported to be significantly negatively correlated with the *Rikenellaceae_RC9_gut_group.* It is worth noting that the *Rikenellaceae_RC9_gut_group* affects the intestinal permeability and oxidative stress and is positively correlated with the lipopolysaccharide in the serum; it has also been reported to be negatively correlated with IL-10 in the colon (Gao et al., 2020). PICRUSt analysis showed that the arachidonic acid and fatty acid synthesis pathways were enriched in the UC mice, indicating that the UC mice had increased lipid metabolism, which might be related to the upregulation of inflammation-related bacteria, such as in *Enterobacteriaceae* and *Proteobacteria* (Tang et al., 2020). The increase in the glutamate levels in the intestine was used as an energy source against oxidative stress. The SFE inhibited oxidative stress and immune–inflammatory in UC mice, which were mainly represented by the restored metabolism of linoleic acid, arachidonic acid, and fatty acid and the decreased expression of related indicators mentioned above.

With the development of UC, the contents of acetic acid, propionic acid, butyric acid, and valeric acid were reduced, but those of isobutyric acid and isovaleric acid showed the opposite trend, which can be attributed to the following reasons. Firstly, the abundance of SFAs-producing probiotics, such as *Ruminococcaceae*, *Roseburia*, and *Desulfovibrio*, were reduced*.* The abundance of *Roseburia*, *Ruminococcaceae*, *Parabacteroides*, which are the main valeric acid-producing bacteria (Wei, 2018), and *Desulfovibrio*, which uses lactic acid and pyruvate as electron donors and uses sulfate ions as terminal electron acceptors to generate the hydrogen sulfide and acetic acid (Flint et al., 2012), were significantly decreased in the UC mice, resulting in the reduction of SCFAs. Secondly, the isobutyric acid and isovaleric acid are transformed from valine, leucine, and isoleucine by their oxidative deamination, decarboxylation, and decomposition, and these transformation processes were increased in wasting diseases (Huang, 1985; Ren and Zhao, 2008). The metabolic function prediction of the intestinal bacteria in UC mice also indicated that the abundance of bacteria involved in the degradation of valine, leucine, and isoleucine degradation increased. Among the SCFAs, the acetic acid and butyric acid play an important role, their decline was correlated with *Ruminococcaceae* and *Roseburia. Ruminococcaceae* plays a prerequisite role in the production of butyric acid and is responsible for the degradation of polysaccharides, including starch, fiber, and xylan, consuming hydrogen to produce acetic acid after the consumption of fiber and carbohydrates. *Roseburia* uses acetic acid as a substrate to produce butyric acid (Morgan et al., 2012; Allaire et al., 2018), which can induce the production of anti-inflammatory factors, such as IL-10 (Morgan et al., 2012; Allaire et al., 2018), and provides 60% energy to the colon epithelial cells through β-oxidation (Ahmed et al., 2016). The host is in a state of high-energy consumption during severe inflammatory response in UC and dysbiosis of intestinal bacterial (Morgan et al., 2012; Hong, 2019). Proteins are decomposed into amino acids and the level of glycogenic amino acids increases while that of SCFAs decreases (Kong and Gao, 2017) in order to cope with the abnormal energy metabolism and nutrition absorption in UC. Therefore, in this study, the enriched metabolism of arginine, histidine, aspartic acid, and glutamic acid, which are the common glycogenic amino acids, enriched the TCA cycle in mitochondria, and the increased abundance of bacteria involved in the mitochondrial oxidative phosphorylation and TCA were observed in UC mice. Furthermore, the increase in the abundance of pathogenic bacteria also had inverse effects on the host metabolism, such as *Helicobacter*, *Bacteroides*, and *Enterobacteriaceae.* The increase in the levels of trimethylamine (TMA) might be attributed to the significant increase in the abundance of these bacteria in our study*,* which metabolized phosphatidylcholine and choline to TMA (He and Chen, 2017; Hoyles et al., 2018). TMA can be absorbed by the liver and converted into TMA oxide (TMAO), which may be a marker of a high-fat diet, intestinal bacterial metabolism, and colon cancer (Xu et al., 2015). These results suggested that the SFE can raise the levels of SCFAs, restore the energy and amino acid metabolism in the UC model by increasing the abundance of beneficial bacteria, and reducing pathogenic bacteria.

UC is accompanied by the disorder of lipid and bile acid metabolisms (Paramsothy et al., 2019). As shown by the result of PICRUSt and metabolomics analysis, the abundance of the bacteria involved in the metabolism of bile acids and the level of free bile acids, such as cholic acid, decreased. However, the level of sulfurized bile acids, such as 3-sulfodeoxycholic acid, increased in the UC mice. The dysfunction of bile acid metabolism might be attributed to the following reasons: the negative feedback regulation of the bile acid receptor FXR weakened when the FXR was expressed in low concentrations in the UC mice, leading to the synthesis of a large amount of bile acid in the liver, while the transporter that regulates the exit of bile acid from the liver into the gallbladder and small intestine is expressed in lower amounts (Sánchez, 2018). Therefore, the bile acid cannot be excreted into the intestine. The conjunct bile acids are decomposed under the influence of the bile salt hydrolase (BSH) after they enter into the small intestine. The bacteria with BSH activity mainly belong to the *Firmicutes*, which account for about 30% of the intestinal microbiota, while the *Bacteroides* account for about 14% (Shi and Yan, 2020). *Lactobacillus* is a typical genus with good BSH activity (Hu, 2020). The changes in the Firmicutes/Bacteroidetes ratio and BSH-producing bacteria in the intestinal tract of the UC mice might be the cause of disorder in the bile acid metabolism; as mentioned earlier, *Desulfovibrio* is a sulfate-reducing bacterium that uses lactic acid and pyruvate as electron donors and uses sulfate ions as terminal electron acceptors to generate hydrogen sulfide and acetic acid (Allaire et al., 2018; Valdes et al., 2018). Furthermore, the epithelium sulfotransferase secreted by the intestinal epithelial cells increased in the UC mice, which intensified the sulfidation reaction of primary and secondary bile acids (Crittenden et al., 2018). The reduced abundance of *Desulfovibrio* and the imbalance between the desulfurization and sulfidation of bile acid in the UC mice might lead to the increase of sulfurized bile acid. The effects of SFE on the bile acid metabolism might be based on adjusting the composition of intestinal microbiota, thereby increasing the expression of FXR and recovering the bile acid metabolic ability of the liver.

Tryptophan (Trp) is an essential nutrient for mammals. Trp and its endogenous metabolites participate in gut immune homeostasis. The microbiota can directly or indirectly regulate the host's endogenous Trp metabolism, while changes in the Trp metabolism can negatively influence the proliferation and diversity of intestinal microbiota (Nowak et al., 2012; Agus et al., 2018; Natividad et al., 2018). Trp metabolism can be divided into three pathways, including the AhR pathway, kynurenine (KP) pathway, and the 5-hydroxytryptamine (5-HT) pathway. The AhR pathway has the closest relationship with the metabolism of gut microbiota. In this study, the AhR ligands, including the indole-3-carboxylic acid, 2-indolecarboxylic acid, indole-3-carboxaldehyde, and 3-methyldioxyidole of the AhR pathway were reduced in the UC mice. The AhR signal is considered to be the main part of the mucosal immune response and has vital functions, maintaining intestinal homeostasis by influencing the renewal of colonic epithelial cells, barrier integrity, anti-inflammation factors, and immune cells (Agus et al., 2018; Natividad et al., 2018). The main bacteria that produce AhR ligands are *Lactobacillus*, *Peptostreptococcus*, and *Clostridium* (Agus et al., 2018; Natividad et al., 2018). These bacteria are also AhR agonists; the changes in these bacteria might cause a decrease in the regulatory capacity of the AhR pathway of the UC mice. In KP pathway, the kynurenic acid (Kna) is increased along the intestinal tract under normal conditions, but its contents were downregulated in the UC mice. Tryptophan is converted into kynurenine (Kyn) and Kna in the meditation of indole amine 2,3-dioxygenase 1 (IDO). The contents of IDO decrease in the sterile and antibiotic-treated mice; however, it is still unclear which bacteria are responsible for the production of IDO (Takamatsu et al., 2013). Kyn and Kna are AhR agonists, and Kna might produce mucosal protection through the G protein–coupled receptor 35 (GPR35) expressed by the epithelial and immune cells (Gao et al., 2018). The weakened activity of IDO regulation by intestinal bacteria and the stimulation of AhR by Kna might cause a decrease in the mediation capacity of the KP pathway. As reported, the impact of altered IDO activity on the degradation of 5-HT is important, as the lower IDO activity leads to both decreased Kyn and increased 5-HT concentrations. In the 5-HT pathway, the level of melatonin, which is a downstream product of 5-HT, increased significantly in the UC mice. The 5-HT pathway was regulated by tryptophan hydroxylase 1 (TpH1); its activation is controversial in IBD. The expression of TpH1 and the level of 5-HT increase in Crohn's disease but UC has shown opposite results (Manocha and Khan, 2012; Li et al., 2014b; Yano et al., 2015; Agus et al., 2018). Some studies suggest that the 5-HT could aggravate intestinal inflammation (Gao et al., 2019; Ma et al., 2020). The increased levels of melatonin are related to the synthesis of 5-HT in a large quantity, which needs further investigations. The SFE regulated immune homeostasis by influencing the interaction among bacteria, Trp, and its derivatives, thereby regulating three pathways of Trp metabolism in varying degrees.

Based on the above results, it is suggested that the SFE can improve UC by regulating the “host–microbe” interaction. According to the co-expression network, interaction network of specific bacteria, and previous studies, *Odoribacter*, *norank_f__Rs-E47_termite_group*, *Rikenellaceae_RC9_gut_group*, *Romboutsia, norank_f__Desulfovibrionaceae*, *unclassified_f__Oscillospiraceae*, *Alistipes*, and *Escherichia–Shigella* are the main pathogenic bacteria of UC (He and Chen, 2017; Xu, 2018; Wong and Yu, 2019). *Lactobacillus*, *Roseburia*, *norank_f__Muribaculaceae*, *Anaerotruncus*, *Candidatus_Saccharimona*, and *Parasutterella* can inhibit inflammation, reduce oxidative stress, and repair the intestinal mucosa by regulating the metabolism of arachidonic acid, tryptophan, bile acid, and energy and promote the synthesis of SCFAs. Although *Candidatus_Saccharimonas* and *Parasutterella* have no direct strong correlations with the differential metabolites, they have a strong regulatory relationship with the pathogenic bacteria of UC. It is speculated that they can play a therapeutic role by specifically regulating the pathogenic bacteria. According to these results, the functions of some bacterial genus have been verified, while that of some in UC have not been verified yet, which is worth closer exploration. The major studies and information of *Lactobacillus*, *Roseburia, norank_f__Muribaculaceae*, *Anaerotruncus*, *Candidatus_Saccharimona*, and *Parasutterella* are listed in Table 1.

**TABLE 1:** The main existing researches about the 6 functional specific bacteria in our study.

Name	Main research	Definition
*Lactobacillus and Roseburia*	1. A large number of studies have shown that *Lactobacillus* and *Roseburia* are typical probiotics, for example, they are SCFAs generation bacteria, related to a lower abundance of pathogenic bacteria, and decreased inflammatory markers; Nascimento et al. (2020)	Probiotics in UC treatment
*norank_f__Muribaculaceae*	1. It is the main genus in warm-blooded animals, such as humans, mice, and guinea pigs; an anaerobic bacterium that grows on host glycan and α-glucan or plant glycan (hemicellulose and pectin); the specific way to support their growth depends on the abundance of enzymes involved in the degradation of particular carbohydrates; some members of *norank_f__Muribaculaceae* can be targeted by the innate immune system; the species of this genus can produce acetic acid, propionic acid, and succinic acid; Ormerod et al. (2016)	Potential probiotic in UC treatment
	2. It can treat intestinal tract osmotic diarrhea, and it is negatively correlated with triglycerides, low-density lipoprotein cholesterol, IL-6, IL-1β, TNF-α, and body weight; these indicators are closely related to inflammatory bowel disease (IBD); Tropini et al. (2018)	It is still uncertain whether the lack of *norank_f_Muribaculaceae* will cause health problems, but our research suggested that *norank_f_Muribaculaceae* does participate in “bacteria–host” interactions that affect homeostasis and inhibit the growth of pathogenic bacteria
	3. Some members of *norank_f_Muribaculaceae* use intestinal mucus polysaccharides as a growth nutrient and occupy a certain living space in the intestine; intestinal symbiotic bacteria that can utilize mucin polysaccharides were identified in mice, and several strains of bacteria including *norank_f_Muribaculaceae* were selected. These strains of bacteria can inhibit the growth and colonization of pathogenic bacteria in the intestine, such as *Flavobacterium difficile*, by occupying ecological space; Pereira et al. (2020)	
*Anaerotruncus*	1. The abundance of *Anaerotruncus* is negatively correlated with the level of cholesterol, which is the upstream metabolite of bile acid; it is a butyric acid-producing bacterium; its abundance was decreased in UC mice; Liu et al. (2018); Koistinen et al. (2019); Chen et al. (2020b)	Potential probiotic in UC treatment
		At present, the relationship between *Anaerotruncus* and bile acid metabolism is still unclear, but in our research, *Anaerotruncus* was found to have more significant positive correlations with free bile acid, especially cholic acid. It is speculated that *Anaerotruncus* can regulate the synthesis of free bile acids in the liver and decomposition of conjunct bile acid
*Candidatus_Saccharimona*	1. *Candidatus_Saccharimonas* (*TM7*) belongs to the phylum *Saccharibacteria*. *Saccharibacteria* is identified as a cellulose-decomposing bacterium that can provide energy for intestinal epithelial cells; Lian et al. (2020)	Potential probiotic in UC treatment
	*2.* Its abundance was decreased in several acute inflammation models, including acute necrotizing pancreatitis and arthritis, and the decrease of its abundance can increase the expression of TNF-α, IL-1β, and IL-17; Takauji et al. (2021)	There are a few kinds of research about *Candidatus_Saccharimonas* in UC; in our study, *Candidatus_Saccharimonas* is positively correlated with *Lactobacillus* and *norank_f_Muribaculaceae*, and negatively correlated with *Escherichia–Shigella* and *norank_f_Desulfovibrionaceae*; its decrease may induce an inflammatory immune response in UC
*Parasutterella*	1. The relative abundance of *Parasutterella* is related to a host of diseases, such as IBD, obesity, diabetes, and fatty liver disease. As a symbiotic microorganism in the intestine, it cannot degrade carbohydrates but can produce succinic acid. This ability is a potential way for it to support the metabolic interactions of species in the intestinal ecosystem, and it can be stably colonized in normal mice without causing an immune response; Koistinen et al. (2019)	Potential probiotic in UC treatment
	2. With the colonization of *Parasutterella* in the intestine, the level of 3-methyldioxyindole, indole-2-carboxylic acid, and indole-3-carboxylic acid increased significantly, while inosine and xanthine decreased significantly, indicating that it can affect the synthesis of AhR ligand and purine metabolism; Ju et al. (2019)	
	3. The expression of sterol-7α-hydroxylase (CYP7A1) in the bile acid synthesis pathway was significantly increased after its colonization, proving that *Parasutterella* can enhance the metabolism of bile acids; Ju et al. (2019)	Although the interaction network of specific bacteria showed that *Parasutterella* does not strongly relate to metabolites, it is negatively correlated with pathogenic bacteria, including *unclassified_f__Oscillospiraceae*, *Alistipes*, and *Rikenellaceae_RC9_gut_group*, especially *unclassified_f__Oscillospiraceae*. According to the existing studies, it is speculated that *Parasutterella* can combine with other bacteria to treat UC

## Conclusion

In conclusion, our previous study found that the SFE had good therapeutic effect on UC rats. This study demonstrated the effects of the SFE against UC by regulating the “host–microbe” interaction. These results demonstrated that the SFE can improve the mucosal damage of the intestine, inhibit immune inflammation, and reduce oxidative stress against UC. These effects are related to improving the dysbiosis of intestinal microbiota, decreasing the abundance of pathogenic bacteria, and regulating the overall metabolic function. These functions might be related to the enrichment of *Lactobacillus*, *Roseburia*, *norank_f__Muribaculaceae*, *Anaerotruncus*, *Candidatus_Saccharimona*, and *Parasutterella* by the SFE.

## Data Availability

The datasets presented in this study can be found in online repositories. The names of the repository/repositories and accession number(s) can be found below: NCBI with accession PRJNA731013 (https://www.ncbi.nlm.nih.gov/bioproject/PRJNA731013).
