# Supplementary material for: Construction of a “Bacteria-Metabolites” Co-Expression Network to Clarify the Anti–Ulcerative Colitis Effect of Flavonoids of Sophora flavescens Aiton by Regulating the “Host–Microbe” Interaction
Source: Front Pharmacol. 2021 Oct 14;12:710052. doi: 10.3389/fphar.2021.710052 (PMC8553221; doi:10.3389/fphar.2021.710052)
Supplement: Supplementary file 1 [file DataSheet1.docx]

Supplementary Material

# Experiment materials

Evans blue and formamide were purchased from Shanghai Macklin Biochemical Technology Co., Ltd; Krebs-Ringer buffer was purchased from Beijing Leagene Biotechnology Co., Ltd; 4 % paraformaldehyde tissue fixative, anhydrous alcohol, methanol, xylene, neutral balsam, HE staining, differentiation solution, bluing buffer, EDTA antigen retrieval solution, PBS buffer, histochemistry kit DAB staining solution, bovine serum (BAS), autofluorescence quencher, DAPI staining solution, and antifade mounting medium were purchased from Wuhan Servicebio Technology Co., Ltd; SAKURA Tissue-Tek® O.C.T. Compound was purchased from Shanghai Lianshuo Biological Technology Co., Ltd; Horseradish peroxidase (HRP) chemiluminescent substrate were purchased from Thermo Scientific Co., Ltd; Skimmed milk powder was purchased from Beijing Solarbio life science Co., Ltd; PVDF membrane was purchased from Merck Co., Ltd; Primary antibody diluent, secondary antibody diluent, BCA kit, RIPA lysis buffer, APS, and TEMED were purchased from Shanghai Beyotime Biotechnology Co., Ltd; 40 %Acr/Bis, 5x loading buffer, Tris-HCl (pH=8.8/6.8), 10x TBST, Trisbase, glycine, SDS, NaOH, and TruSeqTM DNA Sample Prep Kit were purchased from Shanghai Sangon Biotech Co., Ltd.

# Experiment Methods

## UPLC-MS methods for SFE determination

2.1.1. Chromatography

The chromatographic experiments were performed on the UltiMate 3000 UPLC system (Thermo Scientific America). An aliquot of 3 μL of each sample solution was injected into a Hypersil GOLD C18 column (2.1 mm × 100 mm, 1.9 µm) maintained at 40 °C and the flow rate was 0.3 mL/min. The mobile phase consisted of solvent A (0.1 % formic acid in the water, v/v) and solvent B (0.1 % formic acid in acetonitrile). The column was eluted with a gradient of 3 %-25 % B from 0-8 min, 25 %-30 % B from 8-10 min, 30 %-40 % B from 10-18 min, 40 %-55 % B from 18-24 min, 55 %-70 % B from 24-27 min, 70 %-90 % B from 27-28 min, 90 % B from 28-30 min.

2.1.2. Mass spectrometry

The mass spectrometric analysis was performed using a Thermo Q-Exactive Quadrupole Orbitrap spectrometer (Thermo Scientific, America). The ESI source was operated in the negative ion modes. The optimal parameters were as follows, a capillary voltage of 3.0 kV, ion transport tube temperature of 320 °C, collision energy of 15, 35 and 55 %. For the full scan, the data were collected from m/z 100 to 1000. The flow rate of sheath gas flow rate was maintained at 15 L/min and that of auxiliary gas flow rate was 5 L/min.

## Colon permeability evaluation

The surgical thread was used to ligate one end of the colon, and connect the other end to the gavage needle. Injecting 100 μL of 1.5 % Evans blue solution into the intestinal sac, and placing intestinal sac in a centrifuge tube that contained Krebs-Ringer buffer, incubating for 30 min in 95 % O2 and 37 ℃ water bath to maintain activity; The intestinal sac was taken out and flushed with normal saline, and its wet weight was weighed; The intestinal tissue was immersed in 1 mL of formamide, incubating for 24 h in 50 ℃ water bath, and then taken out, the supernatant was obtained. Measuring the absorbance at 655 nm, and calculating the content of Evans blue in the intestinal tissue according to the standard curve.

## Western blot

The concentrations of protein supernatant were determined using a BCA assay kit by the instruction. All samples were diluted with lysate and loading buffer at 4:1, and then denatured at 100 ℃ for 10 min, stored at 20 ℃ for the following separation. The proteins were separated using sodium dodecyl sulfate-polyacrylamide gel electrophoresis (SDS-PAGE), and the preparation of electrophoresis solution and SDS-PAGE were shown in Table S1 and Table S1 respectively. Ten microliters of sample and maker (protein standard) were added into the sample hole, respectively. Electrophoresis was carried out under 80V at constant voltage until bromothymol blue reach the boundary between concentrated gel and separation gel, and then the voltage was adjusted to 120 V, maintained at a constant voltage electrophoresis until bromothymol blue reach the bottom of the gel. The separated proteins were transferred from gel to polyvinylidene fluoride (PVDF) membrane, and then transferred under the constant 300 mA for 120 min, the preparation of transfer solution was shown in Table S1. The PVDF membrane were blocked with 5 % skimmed milk for 1h at room temperature and blotted with primary antibody (1:1000) for 12 h at 4 ℃, followed by secondary antibody (1:10000) for 2h at room temperature. Finally, the PVDF membranes were incubated with horseradish peroxidase (HRP) chemiluminescent substrate, the signals were captured with ECL chemiluminescent system. The ImageJ was used to calculate the gray value of the scanning results.

## Hematoxylin & Eosin (HE)

The tissue was dehydrated, waxed, paraffin-embedded and cut into slice. The following steps were carried out, the slices were dewaxed, they were put into xylene twice for 20 min, rinsed by anhydrous alcohol twice for 5 min, rinsed by 75 % ethanol for 5 min, and rinsed by water; The Paraffin-embedded samples were stained with H&E, the samples were immersed in hematoxylin for 5min, rinsed by water, differentiated with differentiation solution, rinsed by water, stained with bluing buffer, and rinsed by water; The samples were stained with eosin and dehydrated, the samples were rinsed by anhydrous alcohol third for 5 min, rinsed by xylene twice for 5 min, and sealed by neutral balsam.

## Immunofluorescence analysis

CD4 positive inflammatory cell infiltration analysis was performed on paraffin-embedded colon tissue slices. Briefly, the slices were deparaffinized, rinsing the paraffin sections in dimethylbenzene for 15 min for 3 times, rinsing with ethanol for 5 min for 2 times, rinsing with 85 % ethanol for 5 min and 75% ethanol for 5 min, and rinsing with distilled water. The slices were soaked in EDTA antigen retrieval solution, and the antigen was repaired in microwave oven under the following steps, after 8 min of medium fire, cease fire for 8 min, changed to medium-low fire for 7 min, and washed the slices with PBS for 5 min for 3 times. Then they were treated with 3 % BAS at room temperature for 30 min, incubated with the primary antibody at 4 ℃ overnight, and incubated with the secondary antibody at room temperature for 1 h. The slides were then counter-stained with DAPI for 10 min in dark. The reaction was stopped by autofluorescence quencher, and then the images were acquired by microscope.

## Immunohistochemical analysis

The slices were deparaffinized, rinsing the paraffin sections in dimethylbenzene for 15 min for 3 times, rinsing with ethanol for 5 min for 2 times, rinsing with 85 % ethanol for 5 min and 75% ethanol for 5 min, and rinsing with distilled water. The slices were soaked in EDTA antigen retrieval solution, and the antigen was repaired in microwave oven under the following steps, after 8 min of medium fire, cease fire for 8 min, changed to medium-low fire for 7 min, and washed the slices with PBS for 5 min for 3 times. The slices were treated with 3 % hydrogen peroxide for 20 min at room temperature in dark, and then rinsed with PBS for 5 min for 3 times. And then the sections were blocked with 3% BAS for 30 min at room temperature, incubated overnight with the corresponding primary antibody at 4 ℃, and then rinsed with PBS for 5 min for 3 times. For secondary reactions, the sections were incubated with HRP-labelled second antibody at room temperature for 1 h, and then the slices were rinsed with PBS for 5 min for 3 times. DAB was used as chromogen to incubate for 10 min, and the slices were rinsed with water. Hematoxylin was used to counterstain for 3 min, and the slices were rinsed with water. After differentiation, the slices were rinsed with water. Returning blue by bluing buffer and the slices were rinsed with water. The slices were put into 75 % ethanol for 15 min, 85 % ethanol for 5 min, anhydrous ethanol for 5 min for 2 times, xylene for 5 min for 2 times, and sealed by neutral balsam. Finally, microscopic examination and image acquisition analysis were carried out, six fields were selected for each slice under the magnification of 400x to calculate the number of positive cells in IPP.

## The chromatography and mass spectrum conditions of metabolomics analysis

The mobile phase is composed of 0.1 % formic acid–water (A) and 0.1 % formic acid–acetonitrile (B); The injection volume is 5 μL; The flow rate is 0.3 mL/min with the following gradient elution procedure: 0 to 1.5 min, 5 to 50 % B; 1.5 to 3 min, 50 to 55 %; 3 to 7.5 min, 55 to 55 %; 7.5 to 12 min, 55 to 95 % B; 12 to 15 min, 95 to 95% B. The mass spectrometry conditions are as follow, electrospray ionization source and electrospray voltage are 3 kV; ion transfer tube temperature is 320 ℃, sheath gas flow rate is 15 L/min, the auxiliary gas flow rate is 5 L/min, collision energy is 15, 35, and 55 eV, the mass full scan range is *m/z* 50-750 Da.

## The chromatography and mass spectrum conditions of SCFAs determination

One microliter of liquid was injected in split mode with a ratio of 20:1. The solvent delay time was 4 min. The initial oven temperature was 100 °C, and maintained for 3 min, then raised to 150 °C at 5 °C/min, increased to 200 °C at 20 °C/min, finally held at 200 °C for 5 min. Helium was used as a carrier gas at a constant flow rate of 1 mL/min through the column. The temperatures of the injection port, transmission line, and electron impact (EI) ion source were set at 250, 250, and 230 ℃, respectively. The electron energy was -70 eV.

**3 Supplementary Tables and Figures**

**3.1 Supplementary Table**

**Table S1.** Identification of prototypes from SFE in the plasma and urine of rats

| **NO** | **Identification** | **Rt (min)** | **[M-H]-** | **Theoretical Mass (m/z)** | **Measured Mass (m/z)** | **MS/MS fragment** | **Source^1^** |
| --- | --- | --- | --- | --- | --- | --- | --- |
| 1 | (2S)-liquiritigenin | 11.01 | C_15_H_11_O_4_ | 255.0663 | 255.0661 | 119.0502,135.0088,91.0189,153.0193 | U P |
| 2 | Calycosin | 11.48 | C_16_H_11_O_5_ | 283.0612 | 283.0612 | 268.0375,283.0608,211.0406,239.0344 | U P |
| 3 | Trifolirhizin | 11.70 | C_22_H_22_O_10_ | 461.1195 | 461.1194 | 283.0612,254.0588,255.0653 | U |
| 4 | Genistein | 12.82 | C_15_H_9_O_5_ | 269.0455 | 269.0453 | 269.0453,133.0293,201.0555,107.0138 | U P |
| 5 | 8-(3-hydroxymethyl-2-butenyl)-5,7,2’,4’-tetrahydroxyflavanone | 13.64 | C_20_H_19_O_7_ | 371.1136 | 371.1128 | 209.0819,161.02451,179.0712,124.0165 | U P |
| 6 | 2'-hydroxy-isoxanthohumol | 14.57 | C_21_H_21_O_6_ | 369.1344 | 369.1343 | 161.0244,207.1026,135.0452,138.0324 | U P |
| 7 | 3β,7,4’–trihydroxy–5–methoxy–8-prenylflavanone | 15.25 | C_21_H_21_O_6_ | 369.1344 | 369.1343 | 207.1026,138.0232,341.1402,147.0244 | U P |
| 8 | Formononetin | 15.27 | C_16_H_11_O_4_ | 267.0663 | 267.0659 | 252.0425,223.0395,251.0350,132.0218 | U P |
| 9 | 3α,7,4’–trihydroxy–5–methoxy–8-prenylflavanone | 15.53 | C_21_H_21_O_6_ | 369.1344 | 369.1344 | 207.1025,138.032,341.1384,137.0243 | U P |
| 10 | Maackiain | 16.60 | C_16_H_11_O_5_ | 283.0612 | 283.0613 | 283.0607,255.0659,254.0578,240.0433 | U P |
| 11 | 3'-hydroxy-daidzein | 16.60 | C_15_H_9_O_5_ | 269.0455 | 269.0454 | 269.0455,241.0506,197.0608,183.0452 | U P |
| 12 | Isoxanthohumol | 16.94 | C_21_H_21_O_5_ | 353.1394 | 353.1397 | 119.0502,353.1398,233.0818,175.0032 | U P |
| 13 | Isoanhydroicaritin | 17.68 | C_21_H_19_O_6_ | 367.1187 | 367.1187 | 367.1186,297.0404,199.0398,253.0501 | U P |
| 14 | Biochanin | 19.40 | C_16_H_11_O_5_ | 283.0612 | 283.0612 | 268.0375,283.0613,239.0347,211.0398 | U |
| 15 | Kushenol N | 19.92 | C_26_H_29_O_7_ | 453.1919 | 453.1922 | 177.0193,149.0244,275.1650,151.0401 | U P |
| 16 | Kushenol I | 20.60 | C_26_H_29_O_7_ | 453.1919 | 453.1919 | 149.0245,275.1650,177.0193,151.0402 | U P |
| 17 | Kurarinone | 21.32 | C_26_H_29_O_6_ | 437.1969 | 437.1969 | 161.0245,275.1658,137.0241,151.0401 | U P |
| 18 | Kushenol X | 21.60 | C_25_H_28_O_7_ | 439.1762 | 439.1763 | 261.1489,137.0244,177.0194,149.0245 | U P |
| 19 | Kushenol U | 23.44 | C_26_H_29_O_5_ | 421.202 | 421.2022 | 119.0503,163.0039,301.1442,217.0506 | U P |
| 20 | Norkurarinone | 23.99 | C_25_H_27_O_6_ | 423.1813 | 423.1814 | 261.1492,161.0244,137.0244,124.0166 | U P |
| 21 | 2'-methoxykurarinone | 23.78 | C_27_H_31_O_6_ | 451.2126 | 451.2127 | 149.0608,134.0374,163.0037,301.1432 | U P |
| 22 | Kushenol L | 24.65 | C_25_H_27_O_7_ | 439.1762 | 439.1761 | 261.1492,149.0245,177.0193,124.0164 | U P |
| 23 | Xanthohumol | 25.81 | C_21_H_21_O_5_ | 353.1394 | 353.1395 | 119.0502,353.1395,233.0813,175.0037 | U P |
| 24 | Isokurarinone | 26.57 | C_26_H_30_O_6_ | 437.1969 | 437.1968 | 437.1968,287.1286,149.0609,163.0035 | U P |
| 25 | Kushenol E | 27.00 | C_25_H_28_O_6_ | 423.1813 | 423.1814 | 261.1491,161.0244,124.0163,137.0243 | U P |
| 26 | Kuraridin | 27.58 | C_26_H_29_O_6_ | 437.1969 | 437.1969 | 161.0244,137.0244,275.1652,151.0397 | U P |
| 27 | Kushenol A | 27.79 | C_25_H_28_O_5_ | 407.1863 | 407.1873 | 261.1491,137.0230,124.0151,125.0229,193.1587,138.0308,287.1282 | P |
| 28 | Demethylkuraridin | 27.26 | C_25_H_28_O_6_ | 423.18131 | 423.1814 | 261.1491,161.0231,137.0230,193.1587,138.0309,135.0437,219.1380,125.0229 | P |

^1^ In the table, U represents urine and P represent plasma in the source column.

**Table S2** The content of 4 flavonoids determined by UHPLC-MS

| **No** | **Compound** | **Rt** | **Regression line** | **R^2^** |
| --- | --- | --- | --- | --- |
| 1 | Kurarinone | 21.32 | y=151479x+76615 | 0.9995 |
| 2 | Norkurarinone | 23.99 | y=200980x+105220 | 0.9999 |
| 3 | Kushenol N | 19.92 | y=126255x-245527 | 0.9996 |
| 5 | Kushenol L | 24.65 | y = 137200x + 7132 | 0.9998 |

**Table S3** The preparation table of electrophoresis solution and transfer solution

|  | Electrophoresis buffer | Transfer buffer |
| --- | --- | --- |
| Distilled water | 1 L | 800 mL |
| Methanol |  | 200 mL |
| Tribase | 3.03 g | 3.03 g |
| Glycine | 14.40 g | 14.40 g |
| SDS | 1 g |  |

**Table S4** The preparation table of SDS-PAGE

|  | 10 % separation gel (mL) | 5 % concentration gel (mL) |
| --- | --- | --- |
| Deionized water | 11.013 | 4.945 |
| 40 % Acr/Bis | 5.625 | 0.9 |
| Tris-HCl | (pH=8.8) 5.625 | (pH=6.8) 0.9 |
| 10 % APS | 0.225 | 0.072 |
| TEMED | 0.012 | 0.0072 |

**Table S5** Relative standard deviation (RSD) of retention time and peak area of QC samples in positive and negative modes

|  | ESI^+^ |  |
| --- | --- | --- |
| m/z-Rt (min) | Rt-RSD (%) | Area-RSD (%) |
| 458.27125-2.96 | 0.696 | 6.37 |
| 439.27691-4.505 | 0.768 | 6.13 |
| 553.38110-13.27 | 0.232 | 11.3 |
| 439.27691-4.505 | 0.768 | 6.13 |
| 422.20807-7.856 | 0.959 | 6.12 |
| 373.23699-10.365 | 0.201 | 9.25 |
|  | ESI^-^ |  |
| m/z_ Rt (min) | Rt-RSD (%) | Area-RSD (%) |
| 272.23446-12.80 | 0.0318 | 9.14 |
| 392.29175-6.63 | 0.321 | 5.63 |
| 439.26665- 7.814 | 0.455 | 4.32 |
| 488.24332-3.488 | 1.55 | 6.01 |
| 257.06591-1.036 | 1.55 | 14.4 |
| 481.3158-10.514 | 0.228 | 4.58 |

**Table S6** Differential metabolites of the control, model and SFEH groups

| Number | RT | Molecular weight | Metabolites | The trend of change | |
| --- | --- | --- | --- | --- | --- |
| ESI+ | | | | M/C | SFEH/M |
| 1 | 4.03 | 499.29611 | Tauroursodeoxycholic acid | ↓*** | ↑### |
| 2 | 4.63 | 472.25715 | Chenodeoxycholic acid sulfate | ↑*** | ↓# |
| 3 | 3.73 | 416.32788 | Calcitriol | ↑*** | ↓### |
| 4 | 13.39 | 400.33306 | Calcidiol | ↑* | ↓## |
| 5 | 3.64 | 390.27594 | 12-Ketodeoxycholic acid | ↓** | ↑### |
| 6 | 6.85 | 392.29083 | Murocholic acid | ↓*** |  |
| 7 | 14.94 | 384.33800 | Vitamin D3 | ↑* | ↓## |
| 8 | 3.81 | 332.25537 | Adrenic acid | ↓*** | ↑### |
| 9 | 12.24 | 320.23187 | 7-HETE | ↑*** | ↓### |
| 10 | 5.28 | 314.24479 | 12,13-DHOME | ↓*** | ↓### |
| 11 | 12.70 | 299.28164 | Sphingosine | ↑*** |  |
| 12 | 5.28 | 296.23434 | 12,13-EpOME | ↓*** | ↑### |
| 13 | 10.19 | 280.23951 | Linoleic acid | ↓*** | ↑### |
| 14 | 5.28 | 278.22383 | alpha-Linolenic acid | ↓** | ↑### |
| 15 | 2.75 | 270.15733 | Estrone | ↓*** | ↑### |
| 16 | 1.30 | 252.08388 | Deoxyinosine | ↑** | ↓### |
| 17 | 2.65 | 232.12050 | Melatonin | ↑** | ↓## |
| 18 | 1.305 | 219.10996 | Vitamin B5 | ↑** | ↓## |
| 19 | 2.87 | 192.02573 | Citric acid | ↑*** |  |
| 20 | 2.81 | 189.04227 | Kynurenic acid | ↓*** | ↑### |
| 21 | 1.23 | 173.07983 | 2-Oxoarginine | ↑** | ↓## |
| 22 | 1.23 | 161.06842 | Aminoadipic acid | ↑*** |  |
| 23 | 3.04 | 161.04740 | Indole-3-carboxylic acid | ↓*** |  |
| 24 | 1.19 | 145.10999 | 4-Trimethylammoniobutanoic acid | ↑*** | ↓### |
| 25 | 1.25 | 145.08484 | 4-Guanidinobutanoic acid | ↑*** |  |
| 26 | 1.29 | 141.08996 | L-Histidine | ↑*** | ↓### |
| 27 | 1.32 | 138.04186 | Urocanic acid | ↑** | ↓### |
| 28 | 1.17 | 133.03729 | L-Aspartic acid | ↑*** |  |
| 29 | 1.81 | 126.04285 | Thymine | ↑*** | ↓### |
| 30 | 1.29 | 125.08387 | 1-Methylhistamine | ↓*** |  |
| 31 | 1.30 | 122.04703 | Niacinamide | ↑*** | ↓### |
| 32 | 1.30 | 111.04430 | Cytosine | ↑* | ↓## |
| 33 | 1.18 | 59.07421 | Trimethylamine | ↑*** | ↓### |
| ESI- | | | |  |  |
| 34 | 14.02 | 466.31100 | Cholesterol sulfate | ↑** | ↓## |
| 35 | 4.37 | 458.23313 | 3-Sulfodeoxycholic acid | ↑*** | ↓### |
| 36 | 12.23 | 436.35459 | 27-Deoxy-5b-cyprino | ↓*** |  |
| 37 | 12.17 | 434.33886 | 3a,7a,12a-Trihydroxy-5b-cholestan-26-al | ↓*** |  |
| 38 | 13.34 | 432.32318 | 3beta,7alpha-Dihydroxy-5-cholestenoate | ↓*** |  |
| 39 | 13.87 | 420.35952 | 3 alpha,7 alpha,26-Trihydroxy-5beta-cholestane | ↓*** |  |
| 40 | 3.78 | 408.28666 | Cholic acid | ↓*** | ↑### |
| 41 | 6.63 | 392.29175 | Ursodeoxycholic acid | ↓*** |  |
| 42 | 3.03 | 382.19852 | 12-Oxo-20-trihydroxy-leukotriene B4 | ↑*** | ↓### |
| 43 | 11.69 | 368.25305 | Cortol | ↑*** | ↓### |
| 44 | 2.81 | 368.21944 | Prostaglandin G2 | ↑*** | ↓### |
| 45 | 10.78 | 366.24345 | Cortolone | ↑*** | ↓### |
| 46 | 2.84 | 366.20355 | 20-Carboxy-leukotriene B4 | ↑*** | ↓### |
| 47 | 3.07 | 350.20874 | Prostaglandin D3 | ↑*** | ↓### |
| 48 | 4.03 | 334.21392 | Prostaglandin B2 | ↑*** | ↓## |
| 49 | 13.61 | 330.25539 | Docosapentaenoic acid (22n-6) | ↑*** | ↓### |
| 50 | 8.78 | 312.22950 | 13-L-Hydroperoxylinoleic acid | ↓*** |  |
| 51 | 1.07 | 178.04660 | Gluconolactone | ↑*** | ↓## |
| 52 | 1.13 | 175.04701 | N-Acetyl-L-aspartic acid | ↑*** | ↓### |
| 53 | 1.12 | 168.02710 | Uric acid | ↑*** | ↓### |
| 54 | 2.70 | 163.06202 | 3-Methyldioxyindole | ↑*** | ↓## |
| 55 | 2.88 | 161.04646 | 2-Indolecarboxylic acid | ↓*** |  |
| 56 | 1.16 | 148.03588 | 2-Hydroxyglutarate | ↑*** | ↓### |
| 57 | 1.01 | 147.05175 | L-Glutamic acid | ↑*** | ↓## |
| 58 | 1.15 | 146.02016 | Oxoglutaric acid | ↑** | ↓### |
| 59 | 2.91 | 145.05153 | Indole-3-carboxaldehyde | ↓*** | ↑*** |
| 60 | 1.12 | 134.02014 | Malic acid | ↑** | ↓## |
| 61 | 1.15 | 129.04146 | Pyrroline hydroxycarboxylic acid | ↑*** | ↓### |
| 62 | 1.16 | 118.02512 | Succinic acid | ↑*** | ↓### |
| 63 | 1.15 | 102.03036 | 2-Ketobutyric acid | ↑*** | ↓### |
| 64 | 1.16 | 90.03013 | Glyceraldehyde | ↑** | ↓## |

↑ indicates that the compound is up-regulated, ↓ indicates that the compound is down-regulated, and the space indicates that there is no significant difference, or the fold change does not meet the standard.

**Table S7** The physiological and pathological changes in UC mice and the regulation of SFE

|  | The physiological and pathological changes in UC mice | The regulation of SFE in pathology interactions |
| --- | --- | --- |
| Pathological changes | 1. Intestinal bleeding (manifested as blood in the stool)  2. The damage of colon mucosal barrier and structure, its specific situations including the decrease of colonic crypt and goblet cells, the necrosis and shedding of epithelial cells (manifested as atrophy of the colon)  3. The happening of immune inflammation in colon, including lymphocytes are diffusely aggregated in the basal, the number of CD4+ T cell is increased, Th17 cell differentiation, IL-17 signaling pathway, NF-kappa B pathway, TNF signaling pathway etc. are activated (manifested as the swelling of colon)  4. The happening of oxidative stress (manifested as the increased levels of antioxidant factors and the decrease of pro-oxidant factors)  5. The changes of metabolites level in vivo  6. The disorder of intestinal bacteria structure (manifested as the ratio of harmful bacteria to beneficial bacteria is out of balance) | 1. When colon mucosal barrier is destroyed, harmful antigens trigger an immune inflammatory response in lamina propria. SFE can repair mucosal barrier to prevent the trigger of immune inflammation.  2. Immune inflammation can induce the aggravation of oxidative stress, for example, inflammatory factors COX-2 and iNOS can promote oxidative damage. SFE can inhibit immune inflammation and then reduce the level of oxidative stress  3. When the structure of the intestinal bacteria changes, the level of its metabolites in the host will also change accordingly. SFE can adjust the abundance of metabolites and gut bacteria to normal level by regulating "host-microbe" interaction. |
| Physiological change | 1. Weight loss  2. Eating less  3. Mental fatigue  4. Diarrhea | When the pathological conditions are improved by SFE, the physiological state will change accordingly |

# 3.2 Supplementary Figure

**Figure S1** **(A)** Rank abundance of the control, model and SFEH groups at the OTU level; **(B)** Alpha diversity index difference analysis; **(C)** Rarefaction curve of the control, model and SFEH groups; **(D)** Hierarchical cluster analysis of the control, model and SFEH groups; **(E)** The intestinal bacteria typing analysis.

**Figure S2** **(A)** TIC of QC sample in positive and negative modes respectively; **(B)** PCA scores of the control, model, and SFEH groups in positive and negative modes respectively; **(C)** The 200 times response permutation testing of OPLS-DA model in positive and negative modes respectively; **(D)** TIC of the SCFAs in each group.
